# Genetic characterization of varicella-zoster and HIV-1 viruses from the cerebrospinal fluid of a co-infected encephalitic patient, Ghana

**DOI:** 10.1186/s12985-022-01854-7

**Published:** 2022-07-26

**Authors:** Philip El-Duah, Augustina Angelina Sylverken, Michael Owusu, Yaw Ampem Amoako, Richmond Yeboah, Richmond Gorman, Emmanuella Nyarko-Afriyie, Julia Schneider, Terry C. Jones, Joseph Bonney, Titus Adade, Eric Smart Yeboah, Tabea Binger, Victor Max Corman, Christian Drosten, Richard Odame Phillips

**Affiliations:** 1 Institute of Virology, Charité - Universitätsmedizin Berlin, Corporate Member of Freie Universität Berlin, Humboldt-Universität Zu Berlin, and Berlin Institute of Health, Berlin, Germany; 2 Kumasi Centre for Collaborative Research Into Tropical Medicine, Kwame Nkrumah University of Science and Technology, Kumasi, Ghana; 3 Department of Theoretical and Applied Biology, Kwame Nkrumah University of Science and Technology, Kumasi, Ghana; 4 Department of Medical Diagnostics, College of Health Sciences, Kwame Nkrumah University of Science and Technology, Kumasi, Ghana; 5 Department of Medicine, College of Health Sciences, Kwame Nkrumah University of Science and Technology, Kumasi, Ghana; 6 Department of Zoology, Centre for Pathogen Evolution, University of Cambridge, Downing St, Cambridge, CB2 3EJ UK; 7 Department of Medicine, Komfo Anokye Teaching Hospital, Kumasi, Ghana

**Keywords:** Varicella-zoster virus, Chickenpox, HIV, Coinfection, Encephalitis, Ghana

## Abstract

**Background:**

Encephalitis is a serious disease of the brain characterized by prodromal and specific neurological symptoms. HIV infections offer opportunistic viruses, such as Varicella-zoster virus (VZV), the chance to cause encephalitis in patients. There is a lack of information on the genetic diversity of VZV in Ghana and other parts of Africa which requires sequencing and characterization studies to address. The active evolution of HIV-1 in West Africa also requires continuous surveillance for the emergence of new genetic forms.

**Case presentation:**

VZV was detected in the CSF sample of an 11-year-old patient presenting with symptoms of encephalitis by real-time PCR diagnostics. To identify possible unknown aetiological pathogens, next-generation sequencing was performed, and revealed an HIV-1 co-infection.

Alignments of concatenated HIV-1 genome fragments in the *gag*, *pol*, *vif*, *env* and *nef* regions and a near-complete VZV genome were analyzed by Bayesian inference, and phylogenetic trees were generated. The VZV sequence belongs to clade 5 and the HIV-1 sequence is a member of the CRF02_AG predominant circulating recombinant form in Ghana.

**Conclusions:**

Diagnostic tests for CSF HIV would be useful where possible in patients presenting with encephalitis due to VZV and other opportunistic viruses in Kumasi to shed light on the role of HIV in encephalitis cases in Ghana. This report reaffirms the role of the CRF02_AG circulating recombinant form in HIV infections in Ghana and also gives a preliminary genetic characterization of VZV in Kumasi, Ghana.

**Supplementary Information:**

The online version contains supplementary material available at 10.1186/s12985-022-01854-7.

## Background

Encephalitis is a serious disease of the brain characterized by prodromal and specific neurological symptoms [1]. Among the most common viral causes of encephalitis known worldwide are Herpes simplex viruses (HSV), Varicella zoster viruses (VZV), and Enteroviruses [2]. Certain viruses can be opportunistic and take advantage of weakened immunity to cause encephalitis [3]. Human immunodeficiency virus (HIV) infections associated with reduced T cell-mediated immunity present such an opportunity. Elevated levels of HIV RNA in the cerebrospinal fluid (CSF) have also been associated with HIV encephalitis with possible subsequent development into AIDS dementia complex (ADC) [4, 5].

Recombinant HIV subtypes that are found in three or more epidemiologically unlinked individuals are referred to as circulating recombinant forms (CRFs). These CRFs exhibit varied patterns of geographic prevalence [6]. Different subtypes of HIV-1 are known to be endemic in Ghana, however the CRF02_AG circulating recombinant form appears to be predominant [7, 8].

VZV primarily causes chickenpox, and the latent virus can be reactivated under certain conditions, causing shingles [9], and is also one of the common causes of encephalitis [2]. VZV encephalitis can result from primary infection or reactivation [10]. The virus is genetically stable and different genotyping approaches have been used including single nucleotide polymorphism typing and whole genome sequencing [11], however classification is currently via international consensus, presently consisting of seven established clades (designated 1–6 and 9) and two putative clades (designated VII and VIII) [11, 12]. Clade 5 is believed to comprise mostly viruses from Africa and regions with emigrants from Africa [13]. To date, only a handful of CSF-derived VZV isolates have been sequenced and analyzed, and a higher sequence diversity was previously found to occur more frequently in the CSF isolates compared to other body compartments [14]

Infection with HIV increases the risk of developing reactivation of VZV infection with accompanying neurological complications as a result of subclinical movement of the virus into the central nervous system (CNS) [15]. Despite effective antiretroviral therapy to suppress HIV proliferation, the virus can continue to replicate in the CNS, which can lead to drug-resistant strains developing in patients with varying degrees of neurological symptoms [16].

Although the main HIV-1 CRF in Ghana appears to be CRF02_AG, there are a plethora of other CRFs and unique recombinant forms (URFs) in the country [7] and we sought to determine the implicated HIV-1 subtype in the CNS co-infection. There is active evolution of HIV-1 in West Africa which calls for continuous surveillance to monitor the emergence of new genetic forms. Information on VZV viruses from most parts of Africa is lacking but necessary to form a complete epidemiological picture. Given the lack of information on the circulating genotypes of VZV from Ghana, and sub-Saharan Africa, we sought to provide a complete genome and assess the genetic diversity of the detected VZV sequence.

## Case presentation

Here we present the case of a co-detection of HIV-1 and VZV in a CSF sample obtained as part of routine care of a patient presenting with symptoms of encephalitis by real-time PCR diagnostics and subsequent next generation sequencing to determine unknown aetiological pathogens in encephalitic patients.

An 11-year-old male presented to the emergency unit of the Komfo Anokye Teaching Hospital in Kumasi, Ghana, with a 3-day history of fever, chills, general malaise and progressively increasing drowsiness. Two days prior to hospital presentation, the mother had initiated treatment with artemether/lumefantrine for suspected malaria, but his condition did not improve. Initial evaluation showed a febrile patient (temperature 38.4 °C), who was not pale or icteric. He was not dehydrated, and the Glasgow coma score (GCS) was 13/15. The neck was supple and Kernig’s sign was negative. Rapid diagnostic test (RDT) for malaria was negative. An initial diagnosis of partially treated malaria with differentials of encephalitis/ meninigo-encephalitis or septicaemia was made. Appropriate samples including CSF were taken for routine biochemical and microbiological laboratory analysis. Results are shown in Table 1. The initial CSF findings did not identify any bacterial aetiology. A portion of the CSF was sent for routine laboratory diagnostics at the Kumasi Centre for Collaborative Research in Tropical Medicine (KCCR) also in Kumasi. Viral RNA extraction was done using the Qiagen Viral RNA mini kit spin protocol according to the manufacturer's instructions.

**Table 1:** Laboratory findings in the patient

Parameter (unit)	Result (reference range)
CSF analysis	
Appearance	Clear
WBC (per mm^3^)	2, lymphocytes
Glucose (mmol/L)	3.2 (2.8–4.4)
Protein (g/L)	0.3 (0.18–0.45)
Gram stain	No organism seen
Blood and urine analysis	
Random blood sugar (mmol/L)	4.5 (2.5–4.4)
Haemoglobin (g/dL)	14.3 (13.6–18.0)
Total WBC (× 10^3^/µL)	6.1 (4.0–11.0)
Neutrophils (× 10^3^/µL)	4.2 (2–7.5)
Lymphocytes (× 10^3^/µL)	1.37 (1.0–4.5)
Platelets (× 10^9^/L)	286 (140–440)
Blood film	Malaria parasites not seen
Blood culture	No bacterial growth
Urine culture	No growth seen

WBC: White blood cells, mm^3^: Millimetre cube, mmol/L: Millimoles per litre

g/L: Gram per litre, g/dL: Gram per deciliter, µL: Microlitre

Presence of VZV DNA was confirmed by real-time PCR testing using a Herpesvirus Multiplex PCR kit (Tib Molbiol, Berlin Germany) and the LightCycler Multiplex DNA Virus Master (Roche, Penzberg, Germany), which yielded a cycle threshold (Ct) value of 24.35. A high-throughput sequencing (HTS) approach for whole genome sequencing was done using the KAPA RNA Hyper Prep Kit (Roche Molecular Diagnostics, Basel, Switzerland) for library preparation and the 150-cycle NextSeq reagent v3 cartridge (Illumina, San Diego, California, U.S.) according to manufacturer’s instructions. Paired reads from the HTS run were assembled against a reference sequence from GenBank using Geneious prime 2019 (https://www.geneious.com). For classification of the VZV sequence obtained, available complete genomes of VZV on NCBI as of February, 2020 were downloaded and duplicates removed. The sequences were then aligned using the MAFFT algorithm available online at https://mafft.cbrc.jp/alignment/server/. All gaps within the alignment were removed using GapStreeze program v2.1.0 also available online at https://www.hiv.lanl.gov/content/sequence/GAPSTREEZE/gap.htmla to yield a final sequence alignment comprising 236 sequences with 119,892 sites of the genome available. Phylogenetic analysis was done by Bayesian inference using the MrBayes [17] plugin in Geneious prime with a chain length of 1.1 million and a subsampling frequency of 200. A general time reversible substitution model with a gamma distribution and proportion of invariable sites was used for the analysis.

The RDP4 software version 4.97 was used to gather evidence of recombination with the RDP, GENECONV, Bootscan, MaxChi, Chimaera and 3Seq algorithms with a cut off *p*-value of 0.05.

For classification of the implicated HIV virus, an alignment of HIV-1 circulating recombinant forms was downloaded from the Los Alamos HIV sequence database [18] as of February, 2020. HIV-1 sequence fragments from this study were included in the alignment and a single concatenated sequence alignment was generated from the fragment alignments. Redundant copies of the same CRFs were randomly removed to yield a final alignment made up of 56 sequences covering 1663 sites from the *gag*, *pol*, *vif*, *env* and *nef* regions of the genome.

## Outcomes

**Fig. 1:**
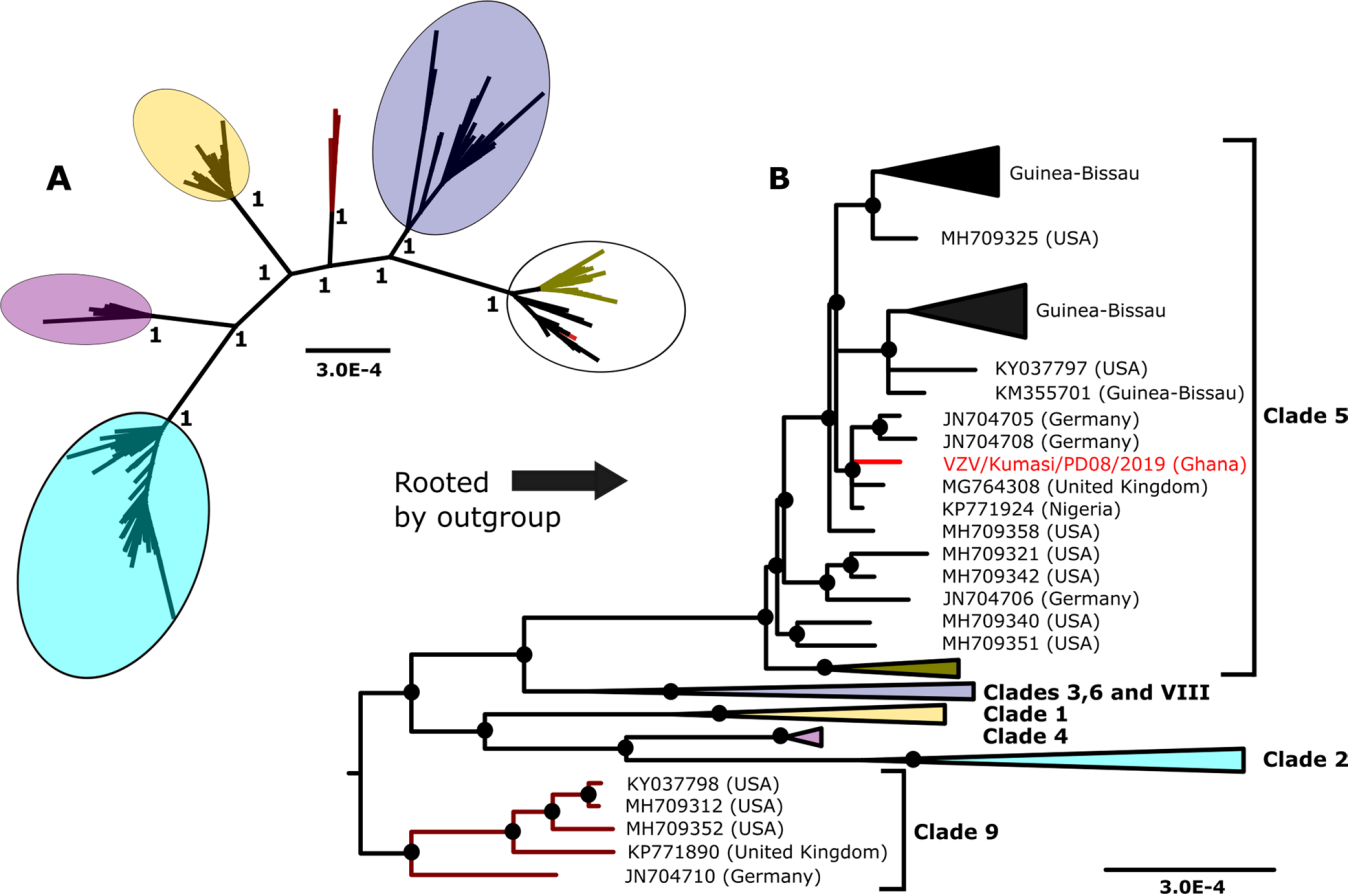
Phylogenetic placement of VZV sequence from co-infected CSF sample. Sequence alignments were done using the MAFFT algorithm and with other sequences from GenBank. Phylogenetic analysis was performed by Bayesian inference using the GTR + G + I substitution model. Sequences in the tree are designated by GenBank accession numbers and origins in brackets with the sequence from this study shown in a red font. Posterior probability support values are indicated at the nodes in the unrooted tree (**A**) and values greater than 0.9 are indicated in the rooted tree by black dots (**B**)

A co-infection with HIV was detected after HTS testing of the VZV positive CSF sample and was determined to be an HIV-1 virus by BLAST analysis. A near-complete genome of VZV which was 123,745 base pairs in length was obtained. The sequence was found to be most closely related to another sequence from Nigeria (GenBank Accession: KP771924) with a pairwise sequence identity of 99.99%. The sequence was submitted to GenBank and assigned accession number OL311042. Phylogenetic analysis showed the sequence to belong to clade 5 which is predominant in Africa (Fig. 1). A recombination event was detected in the sequence by RDP4 which was shared by several other sequences in clade 5 and statistically supported as determined by the GENECONV (*p* = 0.0157), MaxChi (*p* = 0.001) and Chimaera (*P* = 0.0121) methods. There was no major parent identified for this recombination event and may suggest this sequence to be part of a recombinant lineage within the clade with an unknown ancient common ancestor.

## HIV classification

**Fig. 2:**
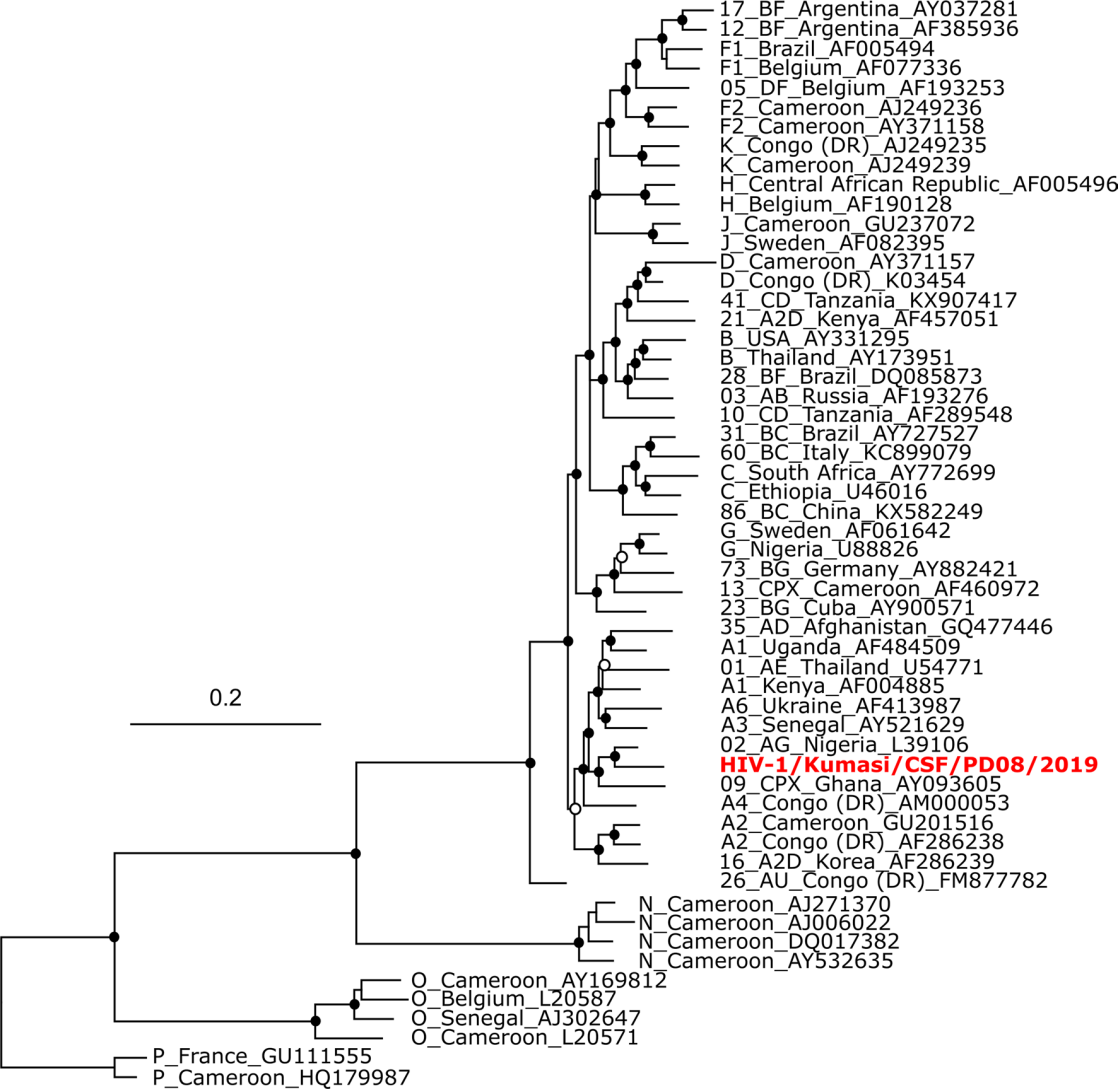
Phylogenetic comparison of circulating recombinant forms of HIV-1. Sequence alignments were done using the MAFFT algorithm and a concatenation of alignments of partial sequences from the *gag*, *pol*, *vif*, *env* and *nef* regions with other sequences from GenBank. Sequences in the tree are designated by GenBank accession numbers and assigned CRF, with the sequence from this study depicted by a red font. Black dots represent notes with posterior probability of 0.90 or greater and white dots represent those greater than 0.75

HIV-1 sequence fragments obtained in the study were related in varying degrees to a variety of circulating recombinant forms with as high as 100% identity in the polymerase gene region (CRF02_AG, Nigeria) and the *nef* gene region (CRFA6, Ukraine) and as low as 77.1% in the envelope glycoprotein gene region (CRFC, Ethiopia). The phylogenetic analysis of the concatenated alignments however found the sequence to cluster with a CRF02_AG circulating recombinant form from Nigeria (Fig. 2). The CRF02 has been found in previous studies to be the dominant strain circulating in Ghana [7, 8]. Sequence data of the short HIV-1 fragments used in this analysis can be found in Additional file 1: Table S1.

## Discussion and conclusions

Accurate diagnosis of CNS infections is essential for timely treatment and improved chances of survival. However, many obstacles hinder accurate diagnoses particularly in resource-limited settings where HIV is also endemic, chief among them the limited availability of laboratory diagnostic tests as well as treatments [19]. In particular, the importance of viral diagnostics becomes more evident in the absence of bacterial etiological agents after routine analysis of CSF following a suspected CNS infection as was the case for the patient in this report.

In settings where opportunistic CNS infections are detected especially in HIV endemic regions, screening for HIV in CSF may be useful. Without accurate diagnoses in the event of co-infection, challenges in treatment occur such as targeted elimination of the detected aetiological agent without consideration of the underlying HIV infection, leading to a poor prognosis. Even in the event of a known prior history of HIV infection and ART treatment, lack of CSF detection as an indicator of compartmentalization and resistance to therapy may hinder considerations for treatment options [20].

During HIV infection, analysis of CSF has been found to be useful for the detection of opportunistic infections like VZV and other co-infections as well as the quantification of HIV viral load in CSF as an indicator for treatment failure and CNS compartmentalization [21]. However, CSF tests and viral load quantification for HIV in encephalitis is not commonly done in the local setting although this would be useful where possible in HIV-positive patients to shed more light on the role of HIV in encephalitis cases.

The VZV sequence obtained in this report unsurprisingly clustered with other clade 5 sequences in line with the predominance of this clade in the African region [13]. A study that investigated VZV viruses from various body compartments found a higher diversity of viruses within CSF from encephalitis patients and hypothesized this to be a result of reactivation from multiple neurons possibly following reinfection and latency with a different strain [14]. This may be likely in areas with high migration from different geographical locations with accompanying different virus strains. Further studies with sufficient CSF-derived VZV strains from different regions will be required to provide a clearer understanding.

Shared recombination events within subsets of sequences within clades as seen in this report may suggest such lineages descended from recombinant parents as suggested by Norbet et al. who assumed this to be the case for intraclade recombination crossovers [22]. This may be useful in further understanding the epidemiology of circulating VZV strains.

The sequence identity of the various sequence fragments in this report hints at a complex recombination history of the detected HIV-1 virus although the sequence appears to belong to the predominant CRF02_AG circulating recombinant form. This does not obviate the need for continuous surveillance to monitor genetic changes that may affect laboratory diagnostics and treatment.

The result of this report provides a useful preliminary genetic characterization of a VZV virus in Kumasi, Ghana and its co-infecting HIV-1 Virus in an encephalitic patient.

## Supplementary Information

**Additional file 1. Table S1.** FASTA sequences of HIV-1 fragments used in phylogenetic analysis.

## Data Availability

All data generated or analysed during this study are available online in publicly available repositories or included in this published article or supplementary files.

## Abbreviations

ADC: AIDS dementia complex
AIDS: Acquired immunodeficiency syndrome
CNS: Central nervous system
CRF: Circulating recombinant form
CSF: Cerebrospinal fluid
GCS: Glasgow coma score
HIV: Human immunodeficiency virus
HSV: Herpes simplex virus
HTS: High-throughput sequencing
KCCR: Kumasi centre for collaborative research into tropical medicine
MAFFT: Multiple alignment using fast fourier transform
NCBI: National center for biotechnology information
PCR: Polymerase chain reaction
RDP4: Recombination detection program version 4
RDT: Rapid diagnostic test
RNA: Ribonucleic acid
URF: Unique recombinant form
US: United States
VZV: Varicella zoster virus
WBC: White blood cells
