# Supplementary material for: Genetic characterization of varicella-zoster and HIV-1 viruses from the cerebrospinal fluid of a co-infected encephalitic patient, Ghana
Source: Virol J. 2022 Jul 26;19:122. doi: 10.1186/s12985-022-01854-7 (PMC9327158; doi:10.1186/s12985-022-01854-7)
Supplement: Supplementary file 1 — Additional file 1. Table S1. FASTA sequences of HIV-1 fragments used in phylogenetic analysis. [file 12985_2022_1854_MOESM1_ESM.docx]

**Additional file 1: Table S1**.

**FASTA sequences of HIV-1 fragments used in phylogenetic analysis**

| >HIV-1/Ghana/PD08/vif3/2019  TAGATCCTGACTTAGCAGACCAACTGATTCACCTGC |
| --- |
| >HIV-1/Ghana/PD08/vif2/2019  CATATTGGGGTCTGCATACAGGAGAAAGAGACTGGCACCTGGGTAATGGGGTCTCCATAGAATGGAGGCAGAAAAACTATAGCACACAA |
| >HIV-1/Ghana/PD08/vif1/2019  ATGGAAAACAGATGGCAGGTGATGATTGTGTGGCAGGTGGACAGGATGAGAATTAGAACATGGA |
| >HIV-1/Ghana/PD08/pol5/2019  GAAGCAGAAGTTATCCCAGCAGAAACAGGACAGGAGACAGCATACTTTATATTAAAATTAGCAGGAAGATGGCC |
| >HIV-1/Ghana/PD08/pol4/2019  TACCAGTTAGAAAAAGACCCCATAGAAGGAGCAGAAACTTTCTATGTAGATGGGGCATCTAATAGGGAGACTAAGCTAGGGAAAGCTGGGTATGTCACTGACAGAGGAAGACAAAAGATTGTTTCCCTAACTGA |
| >HIV-1/Ghana/PD08/pol3/2019  AGTAGCACTGACTGAAGAAGCAGAATTAGAATTGGCAGAGAACAGGGAAATTCTAAAAGAACCTGTACATGGAGCATATTATGATCCAACAAAAGACTTG |
| >HIV-1/Ghana/PD08/pol2/2019  ACCAATATATGGATGATTTATATGTAGGGTCAGACTTAGAAATAGGGCAGCATAGAGCAAAAATAGAGGAACTGAGAGAACATCTACTGAGATGGGGATTTACCACACCAGACAAAAAACATCAGAAAGACCCTCCATTTCTTTGGATGGGGTATGAACTCCATCCTGAC |
| >HIV-1/Ghana/PD08/pol1/2019  AGAAAGTATACTGCATTCACTATACCTAGTATAAATAATGAGACACCAGGGATTAGATATCAGTACAATGTGCTTCCACAGGGATGGAAAGGATCGCCAGCAATCTTTCA |
| >HIV-1/Ghana/PD08/nef3/2019  GGAGCTTTCGATCTCAGCCACTTTTTAAAAGAAAAGGGG |
| >HIV-1/Ghana/PD08/nef2/2019  GAGGAGGTAGGCTTTCCAGTCAGGCCACAGGTACCTTTGAGACCAATGACCTATAA |
| >HIV-1/Ghana/PD08/nef1/2019  GAAGCACAAGAGGAGGA |
| >HIV-1/Ghana/PD08/gag2/2019  CAAGAACAAATAGGATGGATGACAGGCAATCCAGCTATCCCAGTGGGAGAAATATATAAGAGATGGATAGTTCTGGGATTAAATAAAATAGTAAGAATGTATAGCC |
| >HIV-1/Ghana/PD08/gag1/2019  AAATGCATGGGTAAAAGTAATAGAAGAAAAGAATTTTAGCCCAGAAGTAATACCCATGTTTACAGCATTATCAGAGGGAGCCACCCCACAAGATTTGAATATGATGCTAAACATAGTGGGGGGACATCAGGCAGCAATGCAGATGTTAAAAGATACCATCAATGAGGAAGCTGCAGAATGGGACAGAACACATCCAGTCCATGCAGGGCC |
| >HIV-1/Ghana/PD08/Env5/2019  GGGCAAGCCTGTGGAATTGGTTTGACATTTCAAAATGGCTGTGGTATATAAAAATATTCATAATGATAGTAGGAGGC |
| >HIV-1/Ghana/PD08/Env4/2019  AGAGAGAAAAAAGAGCAGTCGGAATGGGAGCGTTACTCCTTGGGTTCTTAGGAGCTGCAGGAAGCAC |
| >HIV-1/Ghana/PD08/Env3/2019  TTCAGGCCTGGAGGAGGAGATATGAGGGACAATTGGAGAAGTGAATTATATAAATATAAAGTAGTAAAAATTGAACCACTAGGTGTAGCACCTACCTATGCAAGGAGAAGAGTGGTG |
| >HIV-1/Ghana/PD08/Env2/2019  CTCAGGAGGAGATGTAGAAATTACAACACATAGTTTTAATTGTGGAGGAGAATTTTTCTATTGCAATACAAC |
| >HIV-1/Ghana/PD08/Env1/2019  GTATCCTTTGAGCCAATTCCCATACATTATTGTGCTCCAGCTGGTTTTGCAATTCTAAAGTGTAATGATAAGGAGTTCAATGGAACAGGGCCATGCAAAAATGTCAGCT |
